# PCR Duplication: A One-Step Cloning-Free Method to Generate Duplicated Chromosomal Loci and Interference-Free Expression Reporters in Yeast

**DOI:** 10.1371/journal.pone.0114590

**Published:** 2014-12-10

**Authors:** Florian Huber, Matthias Meurer, Daria Bunina, Ilia Kats, Céline I. Maeder, Martin Štefl, Cyril Mongis, Michael Knop

**Affiliations:** Zentrum für Molekulare Biologie der Universität Heidelberg (ZMBH), DKFZ-ZMBH Allianz, University of Heidelberg, 69120, Heidelberg, Germany; University of Strasbourg, France

## Abstract

Here, we report on a novel PCR targeting-based strategy called ‘PCR duplication’ that enables targeted duplications of genomic regions in the yeast genome using a simple PCR-based approach. To demonstrate its application we first duplicated the promoter of the *FAR1* gene in yeast and simultaneously inserted a GFP downstream of it. This created a reporter for promoter activity while leaving the *FAR1* gene fully intact. In another experiment, we used PCR duplication to increase the dosage of a gene in a discrete manner, from 1× to 2x. Using *TUB4*, the gene encoding for the yeast γ-tubulin, we validated that this led to corresponding increases in the levels of mRNA and protein. PCR duplication is an easy one-step procedure that can be adapted in different ways to permit rapid, disturbance-free investigation of various genomic regulatory elements without the need for *ex vivo* cloning.

## Introduction

Yeast possesses efficient homologous recombination systems that permit the targeted integration of foreign DNA into the genome [1]–[3]. This enables rapid genomic manipulations, such as gene deletions, plasmid integration or even the induction of complex chromosomal rearrangements [4]. Homologous recombination is highly efficient and works well using short identical sequences of 40–50 bp for targeting the foreign DNA to a specific genomic locus [5]. In combination with marker genes that permit two-way selection - for their presence or their absence - genomic DNA can be modified in many different ways simply by using homologous sequences provided by PCR oligonucleotides. Over the years, a variety of methods and strategies have been developed to achieve virtually any desired genomic modification, from the introduction or removal of sequences to the editing of single nucleotides [6]–[12]. Today, most of these manipulations are based on a plethora of available PCR templates that provide functionality, such as selection markers, protein tags etc. Since the DNA fragments for transformation are made by PCR, this method has been called PCR targeting: a one-step procedure, involving PCR amplification of a generic cassette, the subsequent transformation of the fragment followed by selection of the marker and validation of the correctness of the manipulation. Typically, >80% of the obtained clones are correct. This value depends on the quality of the oligos and the uniqueness of the sequences chosen for homologous recombination.

One-step PCR targeting strategies enable the following genomic manipulations: gene and sequence deletion, C-terminal protein truncation, C-terminal protein tagging, fusion of a heterologous promoter to a gene of interest and N-terminal tagging using heterologous promoters. Large numbers of suitable cassettes are available, see e.g. refs [10], . Recovery of markers for selection can be achieved through a second step and tailored features of the used cassettes. The presence of tandem repeats, for example, enables the spontaneous loop-out of the marker [7], [20], [21], a process which can be further stimulated by endonucleases [17]. Alternatively, Cre- or other recombinases can be used to remove markers, thereby leaving only short sequences behind [16]. Perfect removal of the introduced sequences can also be achieved by a second round of transformation in which a larger fragment of DNA is introduced that contains the desired manipulation (e.g. a single nucleotide exchange) and sequences flanking the marker. Using counter-selectable markers this method was optimized to yield a low rate of false positives, with typically >80% of correct clones [8].

**Figure 1:**
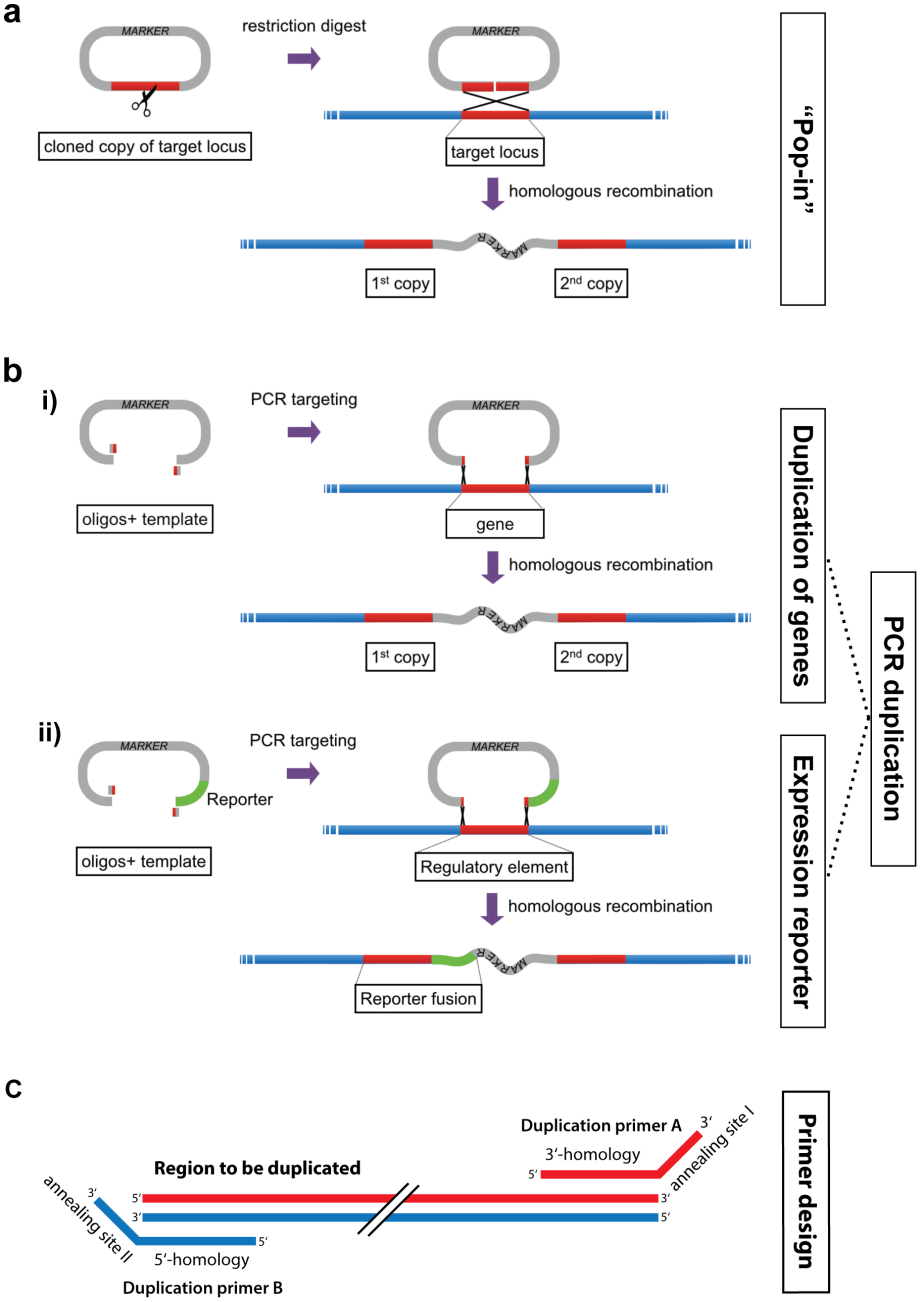
Generation of genomic duplications. (*a*) The ‘Pop-in’ strategy. First, the desired genomic region is cloned into a plasmid containing a marker selectable in yeast. Homologous recombination with the target locus can be stimulated by linearization of the plasmid containing the cloned DNA using a unique restriction site within the cloned fragment. (*b*) The PCR targeting–based duplication strategy (PCR duplication). A PCR product containing a selection marker and short sequences of homology (45–55 bp) to a target locus is transformed into yeast cells. The homologous sequences are provided by the oligonucleotides used for PCR. This leads to the formation of two copies of the target locus, separated by the selection marker. Depending on the template used, PCR duplication can be used for simple duplication of a target locus such as a gene, or it can be used to generate reporter fusions to a specific sequence, e.g. a regulatory element such as a promoter, while a functional copy of the element is retained in its native context. (*c*) Primer design guidelines for PCR duplication. For designing the primers the following rules apply: Duplication primer A = 55 bp from 3′-end of target region in sense orientation + annealing site I. Duplication primer B = 55 bp from 5′-end of target region in antisense orientation + annealing site II.

Yet, no simple cloning-free PCR targeting methods exist that enable the generation of gene duplications or promoter fusions to reporter genes. For locus duplication, Scherer and Davis presented a pioneering method, which also enables seamless genome modification, referred to as ‘Pop-in/Pop-out’ replacement (1979) [6]. There, a two-step procedure is applied. First, the modified allele of the target locus is cloned into a plasmid containing a two-way selectable marker such as *URA3*. The plasmid is linearized by restriction enzyme cleaveage of the plasmid in a sequence homologous to the target locus, followed by transformation into yeast, yielding a duplication of the targeted region (Fig. 1a). However, this method requires *ex vivo* cloning of the initial plasmid.

Expression reporters to monitor the activity of a specific promoter constitute another experimental strategy that may involve the duplication of genomic elements. Here, a regulatory sequence is cloned into a plasmid harbouring a reporter, followed by chromosomal integration. Alternatively, PCR targeting is used to integrate a reporter gene downstream of a regulatory sequence, such as a promoter, thereby disrupting the function of the original gene.

The strategy we present here consists of a single step and avoids cloning of fragments into plasmids or the disruption of gene function. Instead of a cloned full-length DNA fragment for integration, as it is used for ‘Pop-in’ (Fig. 1a), only the outmost 45–55 bp on each side of the target locus are used for targeting the transformed DNA fragment to its genomic locus. These short sequences can be generated by PCR and when targeted to the genome they faithfully produce a duplication of the target locus with the duplicates flanking the amplified selection marker (Fig. 1b). Presumably, this process is mediated by endogenous DNA recombination and repair systems.

**Figure 2:**
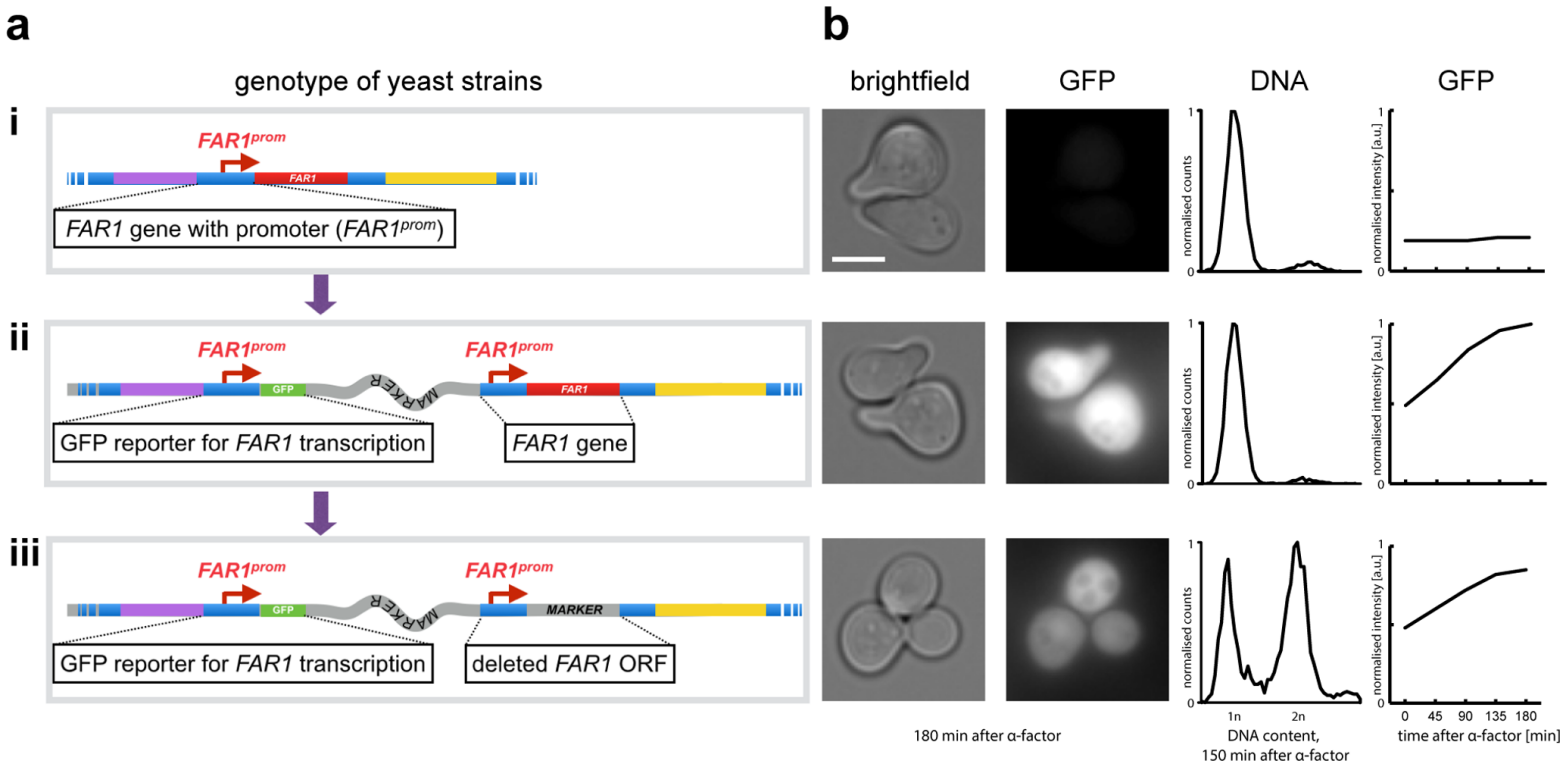
A possible application of the PCR duplication method. (*a*) Generation of genomic promoter-reporter fusions. The PCR duplication strategy was used to obtain from a wild type strain (i) a strain containing GFP fused to the FAR1 upstream intergenic region, including its promoter (FAR1^prom^), plus a copy of the full wild type FAR1 upstream intergenic region and the FAR1 ORF (ii). Subsequently, PCR targeting was used to delete the FAR1 ORF copy left in that strain using a second selection marker (iii). (*b*) Cells from the three different strains (i to iii) shown in (a) were grown to logarithmic phase. After the addition of α-factor (10 µg/ml) samples were removed at the indicated time points and used for microscopy to visualize shmoo formation and quantify GFP fluorescence. DNA content was measured using flow cytometry. Scale bar: 3 µm.

If the target locus encompasses the ORF of a gene along with its 3′- and 5′-regulatory sequences, this leads to gene duplication. If the upstream intergenic region of a gene, which contains its promoter, is targeted and a reporter gene is included in the transformed PCR product, transformation will lead to a duplication of the upstream intergenic region, one of them now controlling the reporter construct and the other one controlling the original downstream sequence. Hence, a promoter reporter is generated while preserving an endogenous version of the promoter and the gene it controls (Fig. 2a). In this work we demonstrate this method using selected examples and two different applications, and we suggest further applications of the method.

## Materials and Methods

### Yeast culturing conditions and strain constructions

Yeast strains used in this study are listed in S1 Table. Cells were cultured according to standard methods [22] at 30°C in either synthetic complete (SC) or YPD medium with antibiotics as required. Strain manipulation procedures were based on PCR-targeting methods as previously described [11], [13]. Cassettes were amplified using a high fidelity DNA polymerase. Plasmids used are listed in S2 Table and oligos used are listed in S3 Table. Strains were validated by colony PCR using Taq DNA polymerase.

### Primer design for duplication of genomic loci

PCR-based duplication of genomic loci was performed by transformation of the PCR product of a pair of primers, which we term “duplication primer A” and “duplication primer B” (Fig. 1c). Conveniently, these primers can be designed in a way that they contain generic annealing sites for PCR amplification of resistance or reporter/tagging cassettes in plasmids with the appropriate binding sites [11], [13], [15]. Upon transformation, the amplified region is inserted in reverse orientation between the duplicated loci. Duplication primer A has a 55 bp overhang that is homologous to the sense strand of the 3′ end of the locus that is to be duplicated. Duplication primer B has a 55 bp overhang that is homologous to the reverse complement of the 5′-end of the target locus. For an illustration of the scheme see Fig. 1c and for detailed design instructions see S1 Methods.

### FACS analysis after pheromone induction

Cells from an overnight culture grown in SC medium were diluted to an OD_600_ of 0.025 (∼2.5×10^5^ cells/ml) and grown to an OD_600_ of 0.4, followed bystimulation with 10 µg/ml of α-factor for 2.5 h. FACS analysis of the DNA content using propidium iodide staining was done as follows: Cells were fixed in 70% ethanol, centrifuged and resuspended in 1 ml of 50 mM sodium citrate containing 0.25 mg/ml DNAse free RNase A (Roche, 10109169001) and incubated for 1 hour at 50°C. 50 µl of 20 mg/ml proteinase K (Merck Millipore, 1.24568.0100) were added followed by another hour of incubation at 50°C. Next, 1 ml of 50 mM sodium citrate containing 16 µg/ml propidium iodide was added. Cells were kept at 4°C overnight prior to FACS analysis using a BD FACS Canto II machine and the FACS Diva v6.1.3 software. 10,000 cells were measured per sample.

### Time course and quantitative light microscopy after pheromone induction

Cells from an SC overnight culture were diluted to an OD_600_ of 0.025 and after reaching an OD_600_ of 0.2 stimulated with 10 µg/ml of α-factor. 20 min before imaging 200 µl of cells were immobilised in 96-well glass bottom plates (Matrical BioScience, MGB096-1-2-LG-L). Time points denote the time after stimulation and were as follows: before stimulation as well as 45, 90, 135 and 180 min after stimulation. Imaging plates were treated with 1∶1000 triethoxysilane aldehyde (UCT Specialties, PSX1055-KG) in 100% ethanol for 15 minutes, washed once with ethanol and water each, treated with 0.1 mg/ml concanavalin A (Sigma, C7555) for 15 minutes and washed twice with water.

Imaging was done with an Applied Precision DeltaVision microscope system consisting of an inverted epifluorescence microscope (IX71, Olympus), a Lumencor Spectra ×LED light source, a 60×NA 1.42 oil immersion objective (Olympus), an sCMOS camera (pco.edge 4.2, PCO) and a temperature-controlled chamber. Eight images were acquired per well from focal plane corresponding to the center of the cells. In addition, imaging of a well containing mCherry-GFP fusions in 1×PBS 5% BSA were taken and served as reference images to correct for uneven illumination. Images without illumination were used to subtract the camera offset from the images. Images were segmented and quantified using ImageJ [23] and custom MATLAB (MathWorks) scripts. For whole-cell segmentation, the Canny edge detection algorithm [24] was used, exploiting the autofluorescence of yeast cells using 390/510 nm (excitation/emission) filter sets.

### RT-qPCR and qPCR

Extraction of genomic DNA (gDNA): 10^7^ cells of a log phase YPD culture were centrifuged for 5 min at 500 g and resuspended in 0.25 ml of S buffer (10 mM K_2_HPO_4_, 10 mM EDTA, 25 µg/ml zymolyase (AMSBIO), pH 7.2), incubated for 30 min at 37°C and then 155 µl of lysis solution (25 mM Tris-HCl, 25 mM EDTA, 2.5% SDS, pH 7.5) were added. 90 µl of 3 M KOAc were added, samples were incubated on ice for 10 min and after centrifugation for 10 min at 16,000 g DNA in the supernatant was recovered by ethanol precipitation. 100 ng of gDNA were used per qPCR reaction. Isolation of RNA: RNA was extracted from 10^7^ cells of a log phase culture in YPD using the EURx GeneMatrix Universal RNA Purification Kit (E3598) according to the manufacturer's instructions. cDNA was synthesised from 2.5 µg of input RNA per sample using M-MLV reverse transcriptase (RT) RNase H^-^ mutant (Promega, M368A) and random hexamers (Thermo Scientific, SO142) according to the manufacturer's instructions. Samples without RT served as negative controls. qPCR runs and quantification: qPCR reactions were performed using a LightCycler 480 II (Roche) with 7.5 µl of LightCycler 480 SYBR Green I Master mix (Roche, 04707516001) and 2.5 µl of either the gDNA solutions or a 1∶5 dilution of the cDNA reaction samples. Primer efficiencies for gDNA and cDNA were determined for all primer pairs by measuring dilution series. Actin was used as a reference gene and relative copy numbers were calculated using the method by Pfaffl [25]. Samples were measured using 5 technical replicates and dilution series using 3 technical replicates. The correctness of the obtained amplification products was verified by melting curve analysis and agarose gel electrophoresis.

### Trichloroacetic acid (TCA) precipitation and Western blotting

Cell lysates for Western blotting were prepared from log phase YPD cultures using the NaOH/β-mercaptoethanol/TCA method as described [13]. For detection of specific proteins, the following antibodies were used: 1∶500 rabbit α-Tub4 (polyclonal, antibody raised against a C-terminal fragment fused to GST, for details see [26]), 1∶5000 mouse α-Pgk1 (monoclonal, Molecular Probes, catalog no.: 459250, clone 22C5D8), 1∶5000 goat α-mouse HRP (polyclonal, Dianova, catalog no.: 115-035-003) and 1∶5000 goat α-rabbit (Jackson Immunoresearch Laboratories, catalog no.: 111035003, lot: 86831). Bands were detected using the Immobilon Western Chemiluminescent HRP Substrate kit (Merck Millipore, WBKLS0500) and an LAS-4000 (Fujifilm) imaging system. Quantification: Western blot images were quantified using ImageJ. Bands were segmented manually and intensities were corrected by subtracting a local background. To correct for unequal loading of lanes intensities were normalized to the Pgk1 signal.

## Results

### A PCR-based ‘Pop-in’ strategy to generate duplicated chromosomal loci

The ‘Pop-in’ strategy of integration of a cloned fragment at the locus homologous to the fragment leads to a duplication of the locus (Fig. 1a). This strategy involves the linearization of the cloned fragment followed by transformation of the fragment into yeast cells. Recombination between the two fragments on either side of the cleavage site then leads to an integration of the fragment and duplication of the locus. In order to avoid cloning of the fragment, we tested if it might be possible to replace the cloning step by a PCR step. Instead of cloning the whole fragment, oligonucleotides that provide short sequences of homology flanking the target locus would be used. This would then result in a duplication of the locus with the duplicates flanking a selection marker. If desired, a tag or any other sequence of choice can be introduced downstream of one of the duplicated loci (Fig. 1b and 1c, for a detailed protocol see S1 Methods).

### Construction of a transcriptional reporter for the *FAR1* gene

In a first experiment to demonstrate this idea, we investigated the possibility to use it to construct a transcriptional reporter. We chose the upstream intergenic region of the *FAR1* gene, which contains the promoter of this gene. *FAR1* is classically used to monitor the activity of the pheromone signalling mitogen-activated protein (MAP) kinase pathway. *FAR1* is induced upon treatment with α-factor and the produced Far1 protein is critically important for cell cycle arrest prior to mating of haploid yeast cells [27], [28]. *FAR1* is not essential for signalling per se, but deletion of this gene leads to a failure in cell polarization and shmoo formation and cells are no longer able to arrest the cell cycle in G1.

We applied PCR targeting using a cassette containing GFP as a reporter gene and *kanMX6* encoding for geneticin resistance as a selection marker. The original purpose of this cassette was to use it for C-terminal GFP fusions using PCR targeting (S1 Table)[11]. The primers prFAR1-dupA and prFAR1-dupB were designed in a way that the resulting PCR product contained 55 bp homologies to the entire *FAR1* upstream intergenic region. This would then yield a fusion of that region with the GFP ORF in the cassette followed by the selection marker (*kanMX6*) and a second copy of the *FAR1* upstream intergenic region and the *FAR1* ORF (Fig. 1b, Fig. 2a and S1 Figure). After PCR targeting, several clones were selected and PCR was used to probe for the presence of the new junctions (S1 Figure). In our experience, correct clones were retrieved at a frequency similar to that of other standard yeast genetic manipulations based on PCR targeting. Pheromone treatment of these new strains led to the expected G1 arrest and shmoo formation, similar to control cells, as shown by microscopy and flow cytometry (Fig. 2b), indicating the presence of a functional *FAR1* gene. Quantification of GFP signals showed a strong increase in GFP intensities (Fig. 2b), indicating the presence of a functional *FAR1* promoter in front of the GFP ORF. As a control we next deleted the *FAR1* ORF in this strain using a second step of PCR targeting and a *natNT2* marker encoding for nourseothricin resistance (S1 Figure) [13]. As expected, this yielded a strain that retained GFP induction upon pheromone treatment, but without showing shmoo formation or G1 arrest, indicative of a failure to arrest the cell cycle [27]. GFP expression was weaker in this strain as cells only respond to α-factor in G1 (Fig. 2b) [29]. This experimentally confirmed that PCR targeting can be used to generate chromosomally localized and disturbance-free expression reporters based upon a local duplication of regulatory sequences.

### Generation of a duplication of the *TUB4* gene

**Figure 3:**
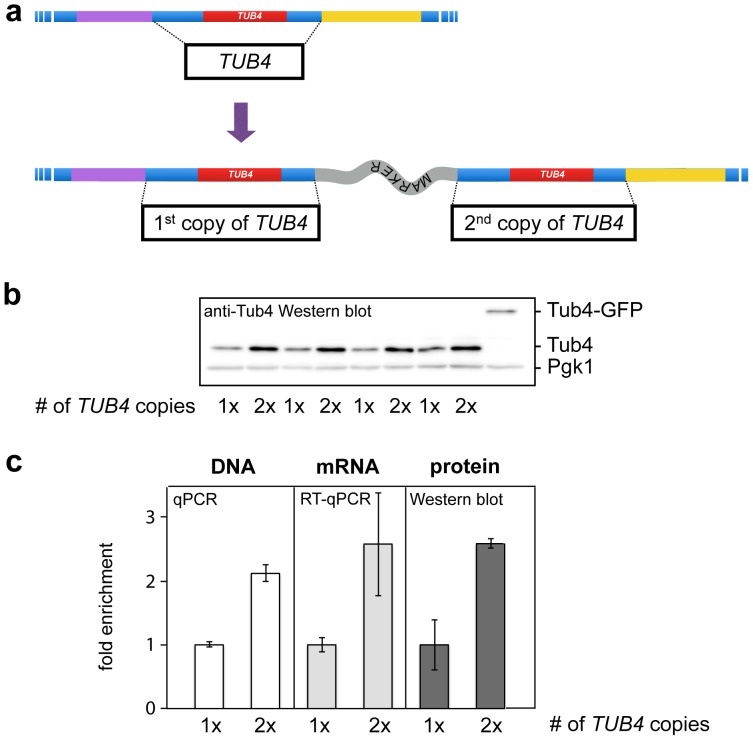
Duplication of the *TUB4* gene. (*a*) PCR duplication of the TUB4 gene including the associated intergenic regions 5′- and 3′- to the TUB4 ORF. (*b*) Western blot and detection of Tub4 using 4 biological replicates of the strain containing the duplication, as well as 4 clones of the wild type strain. Tub4 and, as a reference, Pgk1 were detected using specific antibodies. Quantifications are shown in c. A GFP-tagged version of Tub4 shows the specificity of the antibody. (*c*) Western blot quantification, RT-qPCR and qPCR quantification of TUB4 protein, mRNA and the chromosomal gene copy number, respectively, in the different strains. qPCRs were performed on 1 biological replicate done in 5 technical replicates each. Error bars denote S.D. In all cases, differences between wild type and duplications are statistically significant (two sample t-test, unequal variance, p<0.05).

In a second application, we used our PCR-targeting strategy to generate gene duplications. As an example we chose the *TUB4* gene. This gene encodes the essential γ-tubulin required for microtubule nucleation. Duplication was validated by testing for the newly generated junctions with colony PCR (S2 Figure). Using specific antibodies to detect the protein, we observed a ∼2.5-fold increase in Tub4 protein levels, consistent with the presence of two functional copies of this gene. Likewise, RT-qPCR and qPCR confirmed a corresponding increase in the amount of *TUB4* mRNA and a 2-fold increase in *TUB4* DNA levels (Fig. 3).

## Discussion

Our results show that the ‘PCR duplication’ method presented here is an easy, cloning-free way to generate duplications of genomic loci in yeast. We demonstrate that this method can be used to selectively increase gene dosages and that it enables the simple generation transcriptional reporters in a disturbance-free manner.

For many applications of this method there is no need for the construction of new PCR templates. Instead, published cassettes can be used. For example, any cassette originally constructed for C-terminal gene fusions can also be used to generate an expression reporter. Since a plethora of such cassettes exists (see e.g. refs: [10], [13]–[16], [30], [31]), many different possibilities with respect to reporter genes and selection markers are available. For planning a duplication experiment, care has to be taken to choose the appropriate region for duplication so that, for example, accidental truncation of functional elements is avoided. For this reason the construction of the *FAR1*-expression reporter involved the duplication of the entire *SSY5*-*FAR1* intergenic region, and not only the region spanning the presumed promoter sequence of the *FAR1* gene. Similarly, the duplication of the *TUB4* gene involved the full intergenic regions on the 3′- and the 5′-side of the *TUB4* ORF, as it is possible that regulatory ncRNAs originate outside of the presumed promoter or terminator sequences of an ORF [32], [33].

A critical step in the construction of such genomic duplications is the validation of the correctness of the genomic manipulation. We observed that >80% of the picked clones contained the desired modification as judged by chromosomal PCR on junctions formed upon duplication (S1 and S2 Figures). In principle, this form of genotyping does not discern clones with multiple insertions. Such cases can only be identified by methods such as Southern blotting or qPCR. We investigated the possibility that multiple integration events occur by using a fluorescent protein reporter linked to the marker gene of the PCR cassette. We found that insertion of more than one copy appears to be a rare event: less than 5% of all colonies showed increased levels of fluorescence as compared to the other colonies, which exhibited uniform fluorescence (Matthias Meurer and Michael Knop, unpublished data). Nevertheless, if the exact copy number is critical additional tests to exclude clones with multiple insertions are recommended.

Depending on the experiment, sequencing of the insertions should also be considered due to the possibility of PCR errors or faulty oligos. These may lead to the duplication being associated with a mutation in the sequence used for generating the transformed construct. Experimenters also need to be aware of the fact that recombination between the duplicated loci can lead in rare cases to a spontaneous loss of the duplicated region and the associated cassette. Therefore, we recommend to always maintain selection pressure for the inserted marker.

Finally, we want to note that PCR duplication can be easily adapted for a wider variety of functional studies. In principle, any tag or reporter can be inserted downstream of one of the duplicated loci while preserving the endogenous structure of the other locus. To name just one more example, one could duplicate a gene while simultaneously replacing its C-terminal tag or terminator sequence with another sequence of interest in one of the copies in order to study the effect of that sequence on the expression of the gene. Moreover, we are optimistic that our approach is not limited to yeast but can also be adapted for functional studies in other eukaryotes.

## Supporting Information

S1 Figure **Genomic duplication of gene regulatory regions and genomic reporter gene construction.** (*a*) Construction of strains: a *GFP-kanMX6* cassette [11] is used to generate the duplication of the *SSY5-FAR1* intergenic region and to construct simultaneously a *FAR1*-promoter fusion with the GFP ORF. In a next step the wild type copy of *FAR1* is deleted using a *natNT2* cassette [13]. (*b*) Validation of the strains using PCR and chromosomal DNA (see Materials and Methods) to validate specific new junctions and to confirm the disappearance of other junctions. Strains: *FAR1* → ESM356-1; *pr^FAR1^::GFP::FAR1* → FHY144-1, *pr^FAR1^::GFP::Δfar1* → FHY151-2 Exact genotypes are listed in S1 Table. PCR-A: Far1_j1f & Far1_ur (5′-Junction), Far1_ORF_f & Far1_termrev (3′-Junction); PCR-B: Far1_j1f & Far1_j1r (5′-Junction), Far1_j2f & Far1_j2r (3′-Junction); PCR-C: Far1_j1f & His_and_Kan_Tag2 (5′-Junction), Nat-sense & Far1_termrev (3′-Junction). Oligo sequences are listed in S3 Table.
(TIF)

S2 Figure **Validation of clones carrying** ***TUB4*** **duplications.** Colony PCR was used to validate the new junctions, as indicated in the top panel, using 4 independently obtained clones. Primers for the 5′ and 3′ junctions were Tub4_j1f, Tub4_j1r and Tub4_j2f and Tub4_j2r, respectively. wt =  wild type, ESM356-1. Sizes in bp are indicated on either side of the gel.
(TIF)

S1 Table **Yeast strains.**
(DOCX)

S2 Table **Plasmids.**
(DOCX)

S3 Table **Oligonucleotides.**
(DOCX)

S4 Table **Per well averages of microscopy quantifications of this study.** The underlying images are available from the Dryad Digital Repository: http://doi.org/10.5061/dryad.gj51n.
(XLSX)

S1 Data **FACS raw data.**
(ZIP)

S2 Data **qPCR raw data.**
(ZIP)

S3 Data **Western blot quantifications.**
(ZIP)

S1 Methods **A step-by-step protocol for PCR duplication.**
(DOCX)

S1 References (DOCX)
