# Supplementary figures and images for: PCR Duplication: A One-Step Cloning-Free Method to Generate Duplicated Chromosomal Loci and Interference-Free Expression Reporters in Yeast
Source: PLoS One. 2014 Dec 10;9(12):e114590. doi: 10.1371/journal.pone.0114590 (PMC4262419; doi:10.1371/journal.pone.0114590)

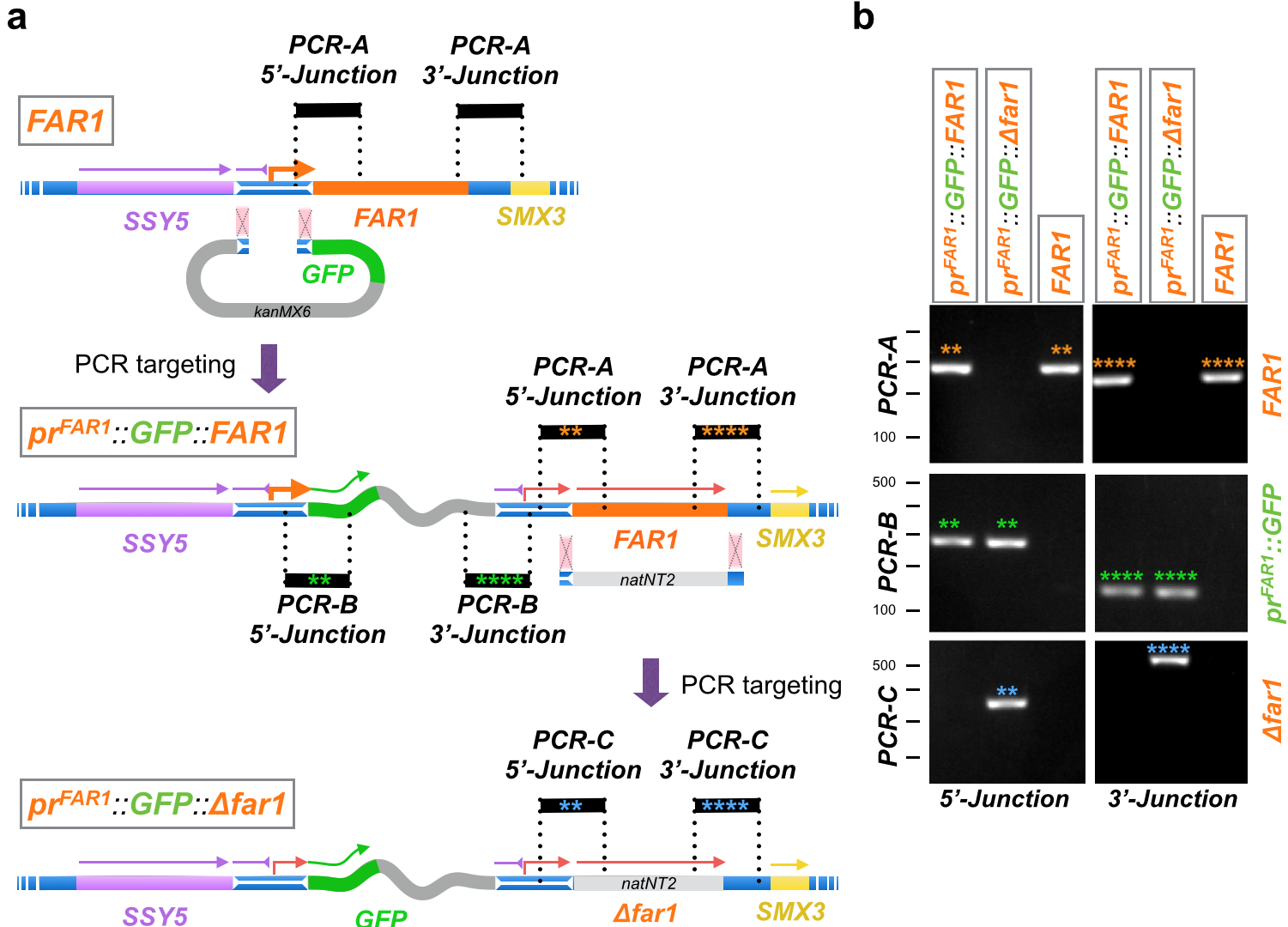

Supplement: S1 Figure — Genomic duplication of gene regulatory regions and genomic reporter gene construction. (a) Construction of strains: a GFP-kanMX6 cassette [11] is used to generate the duplication of the SSY5-FAR1 intergenic region and to construct simultaneously a FAR1-promoter fusion with the GFP ORF. In a next step the wild type copy of FAR1 is deleted using a natNT2 cassette [13]. (b) Validation of the strains using PCR and chromosomal DNA (see Materials and Methods) to validate specific new junctions and to confirm the disappearance of other junctions. Strains: FAR1 → ESM356-1; prFAR1::GFP::FAR1 → FHY144-1, prFAR1::GFP::Δfar1 → FHY151-2 Exact genotypes are listed in S1 Table. PCR-A: Far1_j1f & Far1_ur (5′-Junction), Far1_ORF_f & Far1_termrev (3′-Junction); PCR-B: Far1_j1f & Far1_j1r (5′-Junction), Far1_j2f & Far1_j2r (3′-Junction); PCR-C: Far1_j1f & His_and_Kan_Tag2 (5′-Junction), Nat-sense & Far1_termrev (3′-Junction). Oligo sequences are listed in S3 Table. (TIF) [file pone.0114590.s001.tif]

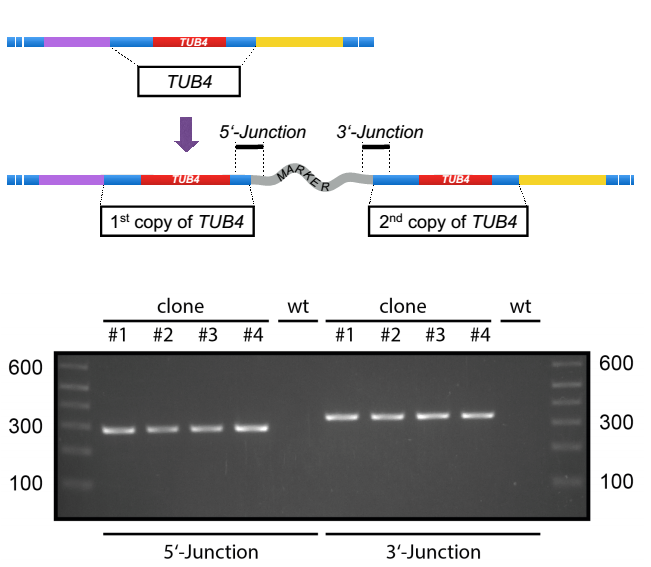

Supplement: S2 Figure — Validation of clones carrying TUB4 duplications. Colony PCR was used to validate the new junctions, as indicated in the top panel, using 4 independently obtained clones. Primers for the 5′ and 3′ junctions were Tub4_j1f, Tub4_j1r and Tub4_j2f and Tub4_j2r, respectively. wt = wild type, ESM356-1. Sizes in bp are indicated on either side of the gel. (TIF) [file pone.0114590.s002.tif]

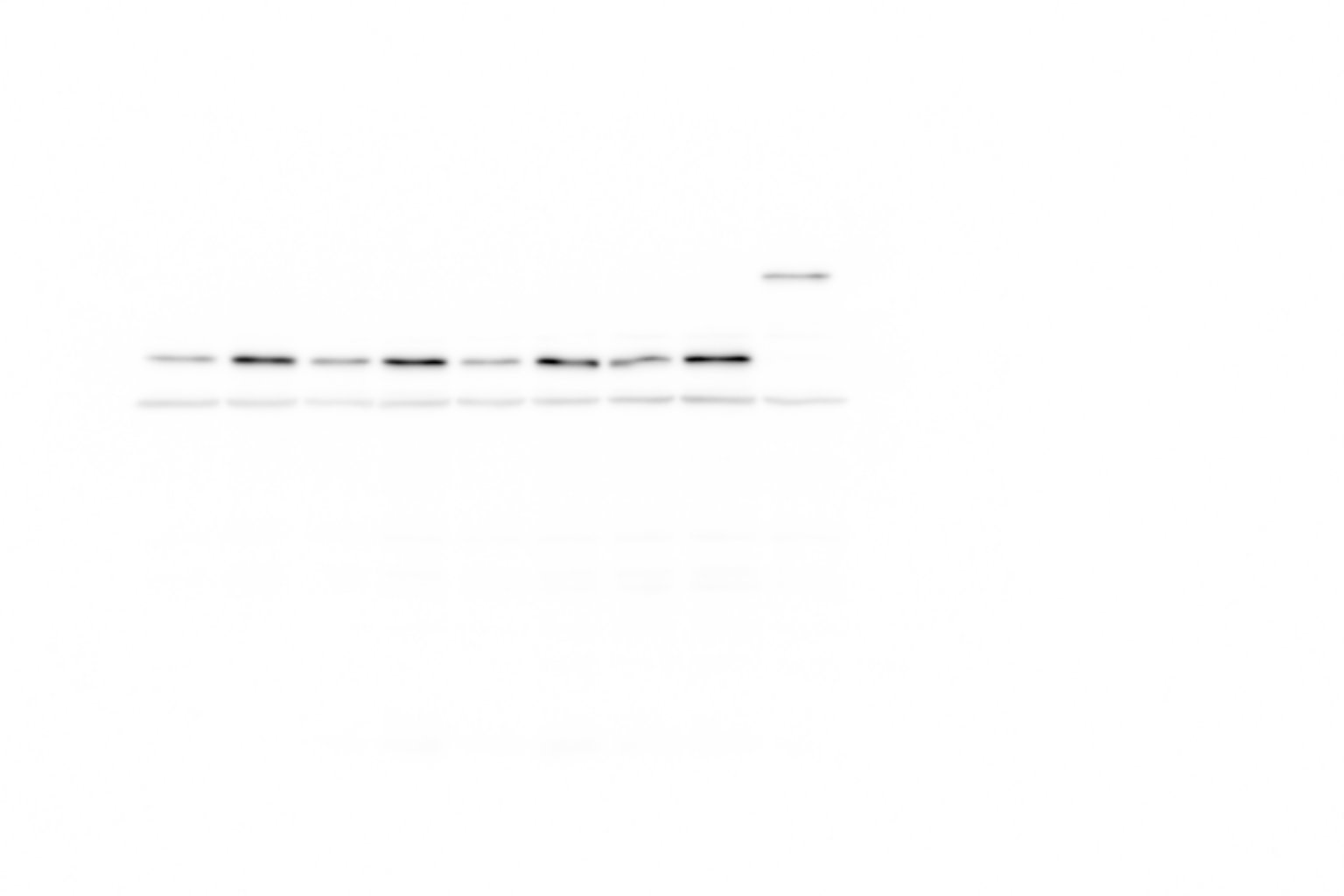

Supplement: S3 Data — Western blot quantifications. (ZIP) [file pone.0114590.s009.zip › Western/2014_05_07_BlotB_PgkTub4_30s.tif]
