# Supplementary material for: PCR Duplication: A One-Step Cloning-Free Method to Generate Duplicated Chromosomal Loci and Interference-Free Expression Reporters in Yeast
Source: PLoS One. 2014 Dec 10;9(12):e114590. doi: 10.1371/journal.pone.0114590 (PMC4262419; doi:10.1371/journal.pone.0114590)
Supplement: S1 Table — Yeast strains. (DOCX) [file pone.0114590.s003.docx]

**Table S1**: Yeast Strains

Name Parent Strain Relevant Genotype Reference

ESM356 S288C Mat**a** *ura3-53* *leu2Δ1 trp1Δ63 his3Δ200* References S1 [35]

FHY144 ESM356-1 *pr^FAR1^::GFP-kanMX6::FAR1* this study

FHY151 FHY144 *pr^FAR1^::GFP-kanMX6::Δfar1::natNT2* this study

FHY150 ESM356-1 *TUB4::hphNT1::TUB4* this study

DHY008 ESM356-1 *TUB4-GFP-kanMX6* this study
