# Supplementary material for: PCR Duplication: A One-Step Cloning-Free Method to Generate Duplicated Chromosomal Loci and Interference-Free Expression Reporters in Yeast
Source: PLoS One. 2014 Dec 10;9(12):e114590. doi: 10.1371/journal.pone.0114590 (PMC4262419; doi:10.1371/journal.pone.0114590)
Supplement: S2 Table — Plasmids. (DOCX) [file pone.0114590.s004.docx]

**Table S2**: Plasmids

Plasmid Backbone Description Reference

pFA6a Plasmid for *E. coli*, *ampR* resistance gene [10]

pMaM4 pFA6a sfGFP, *kanMX6* References S1 [34]

*pFA6a-natNT2* pFA6a *natNT2* cassette [13]

*pFA6a-hphNT1* pFA6a *hphNT1* cassette [13]
