# Supplementary material for: PCR Duplication: A One-Step Cloning-Free Method to Generate Duplicated Chromosomal Loci and Interference-Free Expression Reporters in Yeast
Source: PLoS One. 2014 Dec 10;9(12):e114590. doi: 10.1371/journal.pone.0114590 (PMC4262419; doi:10.1371/journal.pone.0114590)
Supplement: S3 Table — Oligonucleotides. (DOCX) [file pone.0114590.s005.docx]

**Table S3**: Oligonucleotides Annealing sites to plasmids are underlined. Regions of homology are highlighted in grey.

Primer Sequence

**Oligos for strain construction**

prFAR1-dupB GCAATGTCTTTTTTTTTTCATCCTGTAATGGGTTAAATAACTTCAAAAAGGCAAAATCGATGAATTCGAGCTCG

prFAR1-dupA ACAAAGTCTATAGATCCACTGGAAAGCTTCGTGGGCGTAAGAAGGCAATCTATTAATGTCCAAGGGTGAAGAGC

TUB4-dupB GGAATTAGAGGATCCGTTACGGAAGAGAGAACTTCGTAAGACATTATTGCTGTGACGTACGCTGCAGGTCGAC

TUB4-dupA GCTGCACGGATTAGTTCGATAATTCAACGACAGTTATACTAATCTTGGTGATGGAATCGATGAATTCGAGCTCG

S1-FAR1 AAGTCTATAGATCCACTGGAAAGCTTCGTGGGCGTAAGAAGGCAATCTATTAATGCGTACGCTGCAGGTCG

S2-FAR1 AAAAAAAAGGAAAAGCAAAAGCCTCGAAATACGGGCCTCGATTCCCGAACTACTAATCGATGAATTCGAGC

S2-Tub4 CTATAATAAAACTATTGGGCGGTGGTAAAATTCCTGAACAAGGAAGGCATCATTAATCGATGAATTCGAGCTCG

S3-Tub4 TATGGTTGGCGAGTTGGAAGAGGACCTGGATGCCGACGGTGATCATAAATTAGTACGTACGCTGCAGGTCG

**Validation Oligos**

Far1_j1f GAAGGTACCTTGGTTCACAG

Far1_ur TCACGATCTCCACTTGGTG

Far1_ORF_f CGTCTAATTATGGATGAACACC

Far1_termrev ACATTAACTGCTATTTACGACG

Far1_j1r TATGTCCGTTTACATCTCCG

Far1_j2f AATGCTGGTCGCTATACTGC

Far1_j2r TAGTCGCTCCTACAGTGATG

His_and_Kan_Tag2 GAAGGTACCTTGGTTCACAG

Nat-sense GCAGGCGCTCTACATGAGCA

Tub4_j1f GATTCAACTCAGCGTAATGG

Tub4_j1r ACAACCTGAAGTCTAGGTCC

Tub4_j2f GCACGTCAAGACTGTCAAGG

Tub4_j2r CAAAGAGAGAACGTTATAATGG

**qPCR Oligos**

Tub4_rt_f ACCCTTCCAATTCTCTTGTCTC

Tub4_rt_r GCGATATCTGACGAGGTTCTAC

Actin_rt_f GATTCCGGTGATGGTGTTACTC

Actin_rt_r TCAAATCTCTACCGGCCAAATC
