# Supplementary material for: PCR Duplication: A One-Step Cloning-Free Method to Generate Duplicated Chromosomal Loci and Interference-Free Expression Reporters in Yeast
Source: PLoS One. 2014 Dec 10;9(12):e114590. doi: 10.1371/journal.pone.0114590 (PMC4262419; doi:10.1371/journal.pone.0114590)
Supplement: S1 Data — FACS raw data. (ZIP) [file pone.0114590.s007.zip › FACS/FlowJo_Analysis.pdf]

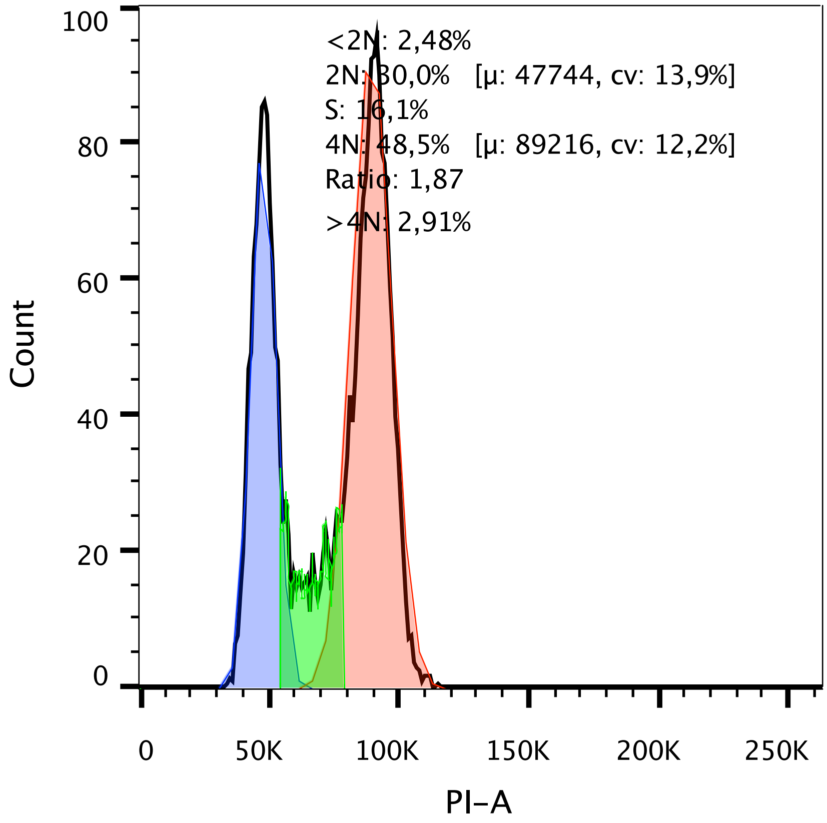

Specimen_001_Tube_001.fcs
Cell Cycle
7533

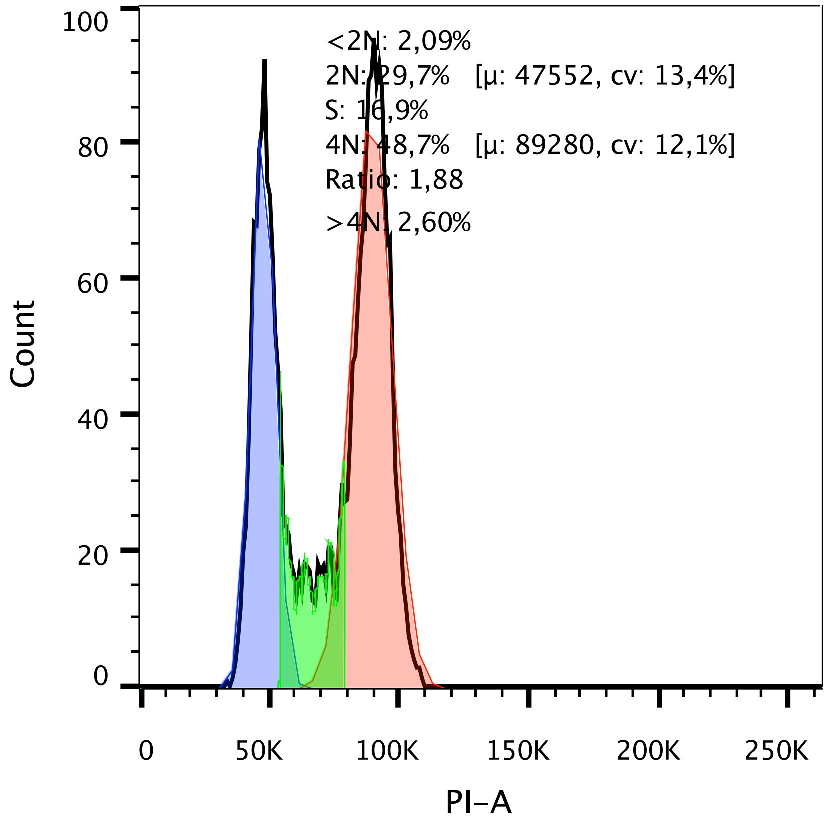

Specimen_001_Tube_002.fcs
Cell Cycle
7660

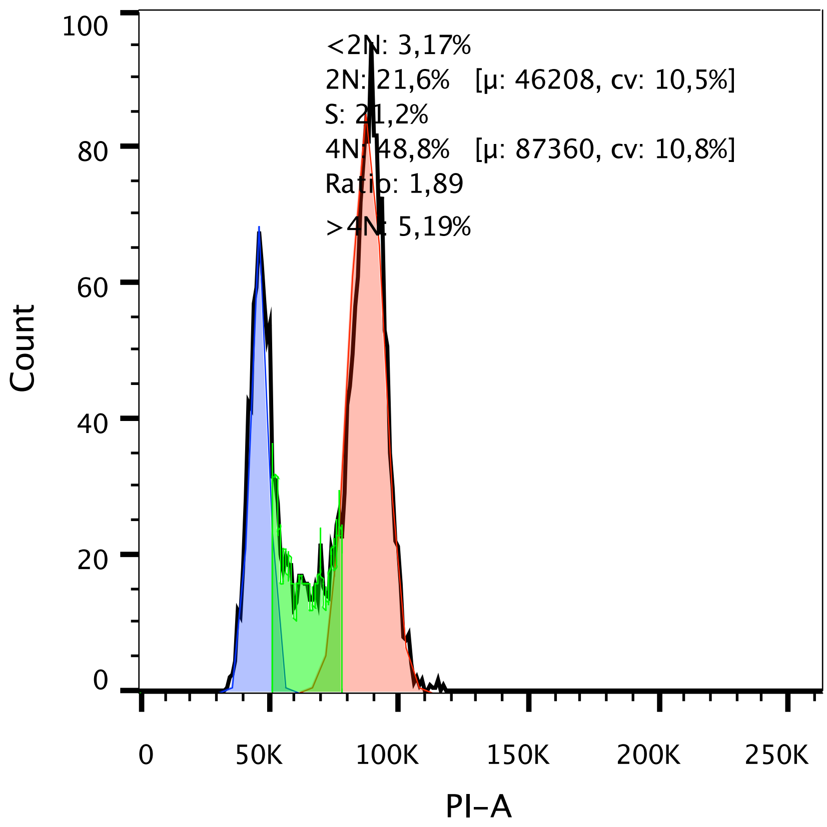

Specimen_001_Tube_003.fcs
Cell Cycle
7033

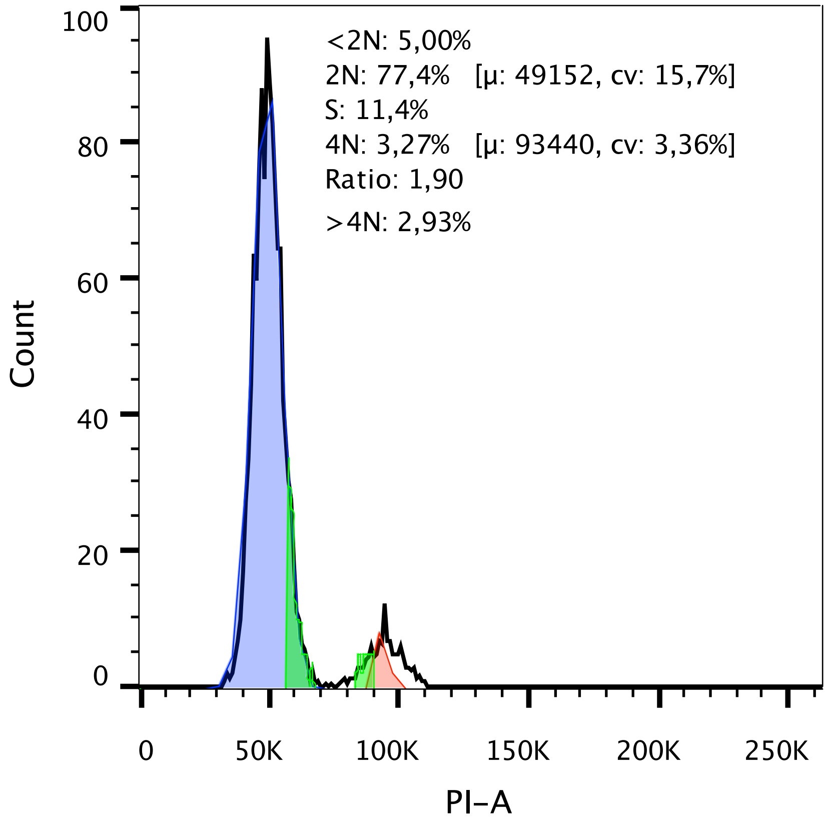

Specimen_001_Tube_004.fcs
Cell Cycle
4159

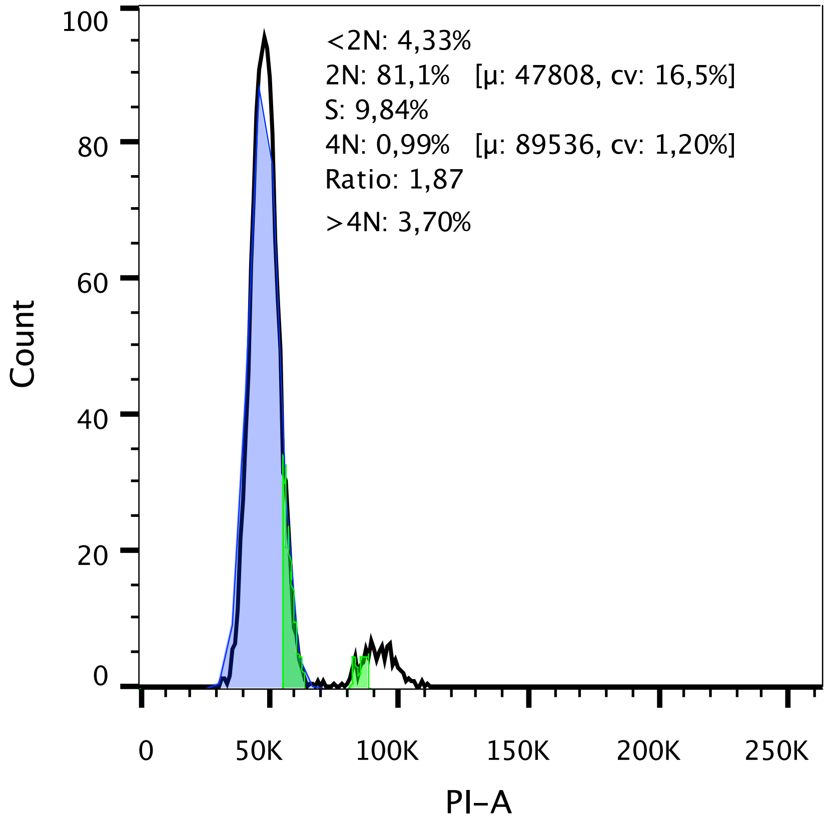

Specimen_001_Tube_005.fcs
Cell Cycle
4245

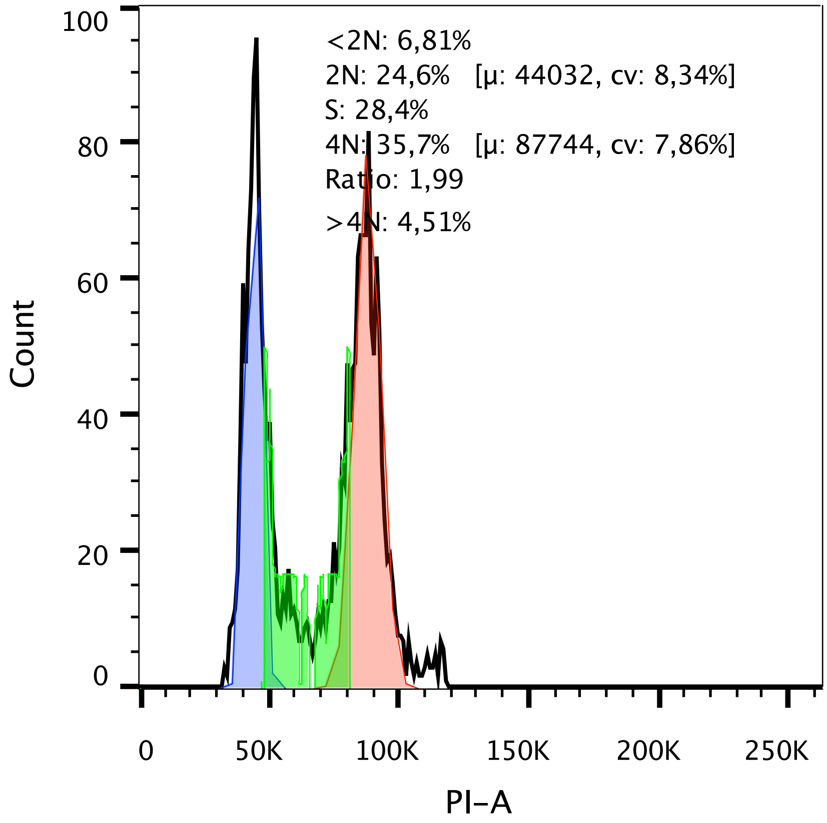

Specimen_001_Tube_006.fcs
Cell Cycle
2083
