# Supplementary material for: PCR Duplication: A One-Step Cloning-Free Method to Generate Duplicated Chromosomal Loci and Interference-Free Expression Reporters in Yeast
Source: PLoS One. 2014 Dec 10;9(12):e114590. doi: 10.1371/journal.pone.0114590 (PMC4262419; doi:10.1371/journal.pone.0114590)
Supplement: S1 Methods — A step-by-step protocol for PCR duplication. (DOCX) [file pone.0114590.s010.docx]

**Methods S1: A step-by-step protocol for PCR duplication**

For PCR duplication of a chromosomal region in yeast the following steps have to be executed (for a visual representation, see also Figure 1b and c:

**1. Determination of the chromosomal region to be duplicated.**

The chromosomal region to be duplicated can range from a short region (e.g. a few hundred bp; the minimal size needs to be determined yet) up to a few kbp. Larger regions may be possible, but we have not tested sequences >10 kb. The region should be chosen such that unintentional interferences with genetic elements in the chromosomal neighborhood are avoided as much as possible. For a more detailed discussion of gene or promoter duplications please refer to the main text.

**2. Selection of a template plasmid**

In *S. cerevisiae*, typical marker cassettes contain antibiotic resistance auxotrophy markers. For a range of cassettes with generic annealing sites for PCR primers please refer to the following publications, among others: [10,11,13-15].

**3. Design of oligonucleotides**

Duplication primer A: Paste 55 bp from the 3’-end of the region to be duplicated in sense orientation + annealing site I in the plasmid.

Duplication primer B: Paste 55 bp from the 5’-end of the region to be duplicated in antisense orientation + annealing site II in the plasmid.

Note that for the construction of reporter fusions it is critical to design a functional linkage between the genomic region and the reporter gene.

**4. PCR amplification of the template cassette**

Run a PCR to generate the DNA molecules to be transformed. Use a high fidelity polymerase to minimise the chance of mutations. For detailed protcols, please refer to the manual of your DNA polymerase or the following references: [11,13].

**5. Yeast manipulations**

5 µl of the PCR product is used to directly transform competent yeast cells according to standard procedures [13]. Wait for colonies to appear and streak them again to single colonies.

**6. Validation of strains**

Genotype the resulting strains to ensure the correctness of the gene duplication by running a chromosomal PCR on the 2 newly generated junctions and the untransformed strain as a negative control (for an example see Figure S1). Keep in mind that the orientation of the marker and an optional tag inserted between the duplicated sequences will follow the PCR product starting from duplication primer A. Moreover, consider that it is possible that multiple copies are integrated into the genome, albeit with low frequency (see also main text).
