# Supplementary material for: PCR Duplication: A One-Step Cloning-Free Method to Generate Duplicated Chromosomal Loci and Interference-Free Expression Reporters in Yeast
Source: PLoS One. 2014 Dec 10;9(12):e114590. doi: 10.1371/journal.pone.0114590 (PMC4262419; doi:10.1371/journal.pone.0114590)
Supplement: S1 References — (DOCX) [file pone.0114590.s011.docx]

**References S1**

34. Gibeaux, R., Politi, A. Z., Nédélec, F., Antony, C. & Knop, M. Spindle pole body-anchored Kar3 drives the nucleus along microtubules from another nucleus in preparation for nuclear fusion during yeast karyogamy. *Genes Dev.* **27,** 335–349 (2013).

35. Knop, M. & Schiebel, E. Receptors determine the cellular localization of a gamma-tubulin complex and thereby the site of microtubule formation. *EMBO J.* **17,** 3952–3967 (1998).
